# Atorvastatin inhibits glioma glycolysis and immune escape by modulating the miR-125a-5p/TXLNA axis

**DOI:** 10.1186/s41065-024-00349-5

**Published:** 2024-12-26

**Authors:** Kang Gao, Tao Zhou, YingChun Yin, XiaoJie Sun, HePing Jiang, TangYue Li

**Affiliations:** 1 Department of Neurosurgery, Central Hospital of Zibo, Zibo City, Shandong Province 255000 China; 2 Department of Pathology, Central Hospital of Zibo, No.54, Communist Youth League West Road, Zhangdian District, Zibo City, Shandong Province 255000 China

**Keywords:** Glioma, Atorvastatin, miR-125a-5p, TXLNA, Glycolysis, Immune escape

## Abstract

**Background:**

Conventional treatments, including surgery, radiotherapy and chemotherapy, have many limitations in the prognosis of glioma patients. Atorvastatin (ATOR) has a significant inhibitory effect on glioma malignancy. Thus, ATOR may play a key role in the search for new drugs for the effective treatment of gliomas.

**Methods:**

U87 cells were treated with different doses of ATOR and transfected. Viability was assessed using MTT, proliferative ability was determined using the colony formation test, Bax and Bcl-2 were identified using Western blot, apoptosis was identified using flow cytometry, and U87 cell migration and invasion were detected using the Transwell assay. Glucose uptake, lactate secretion, and ATP production in U87 cell culture medium were quantified. The positive rates of IFN-γ and TNF-α in CD8T were measured through flow cytometry. Subcutaneous injection of U87 cells was carried out to construct an in vivo mouse model of gliom, followed by HE staining to assess the effects of ATOR and miR-125a-5p on tumor development.

**Results:**

ATOR blocked the viability, proliferation, migration, and invasion of U87 cells through the miR-125a-5p/TXLNA axis, and suppressed glycolysis and immune escape of glioma cells. Furthermore, overexpressing miR-125a-5p enhanced the anti-tumor effect of ATOR in vivo.

**Conclusion:**

ATOR blocks glioma progression by modulating the miR-125a-5p/TXLNA axis, further demonstrating that ATOR provides an effective therapeutic target for the treatment of glioma.

## Introduction

Gliomas rank as the primary cancer in the central nervous system [1], linked to elevated death rates and unfavorable outcomes [2]. Presently, primary treatments for gliomas encompass chemotherapy, surgical procedures, radiotherapy, and immunotherapy [3–5]. Nonetheless, the widespread and hostile characteristics of gliomas, along with the brain tumor barrier, pose substantial obstacles to both pharmaceutical and systemic treatments [6].

Statins impede the reductase activity of 3-hydroxy-3-methylglutaryl-coenzyme A, an enzyme that is essential for cholesterol production [7]. Atorvastatin (ATOR) is widely used clinically for the treatment of hypercholesterolaemia. Recent studies have demonstrated that AVT has a significant inhibitory effect on tumor cells [8–10] and pleiotropic effects on various cellular processes, including apoptosis, angiogenesis, inflammation, senescence, and oxidative stress [11]. Researchers have shown that ATOR can inhibit glioma cell invasion [12].

There has been increasing interest in miRNA’s role in tumor immunomodulation as a non-coding RNA that binds directly to target genes’ 3’ untranslated regions (UTR). miR-125a-5p is aberrantly expressed in human tumors [13] and inhibits proliferation and EMT in gastric cancer [14]. Recently, miR-125a-5p has been measured to be down-regulated in gliomas to inhibit tumor malignancy [15]. TXLNA (Taxilin α), a binding partner of the syntaxin family of proteins, is thought to be a key factor in coordinating intracellular vesicle transportation [16]. TXLNA is higher in glioma cells [17].

Elevated glycolysis is a key factor in tumorigenesis. Unlike normal cells, even under well-oxygenated conditions, tumor cells preferentially use aerobic glycolysis to produce energy [18]. Therefore, tumor growth can be inhibited by suppressing glycolysis levels. In glioma cells, PKM2 expression is significantly increased [19]. PKM2, in addition to regulating glycolysis directly, also regulates other glycolysis-related genes, as well as malignant activities of gliomas [20]. Tumor cells rely on a number of mechanisms to avoid recognition and attack by the immune system during growth and metastasis. An important element of tumor immune escape and immunotherapy is the binding of programmed cell death-1 (PD-1) on immune cells to programmed death ligand 1 (PD-L1). T-cells are inhibited from killing tumor cells by PD-L1 by interacting with the PD-1 receptor on their surface [21, 22]. Therefore, the PD-1/PD-L1 axis is the most rate-limiting step in the anticancer immune response.

The aim of this study was to investigate the effects of ATOR on glioma cell biological functions, glycolysis levels and immune escape via the miR-125a-5p/TXLNA axis. On the basis that ATOR has some therapeutic effect on the progression of glioma, this study hopes to provide new drug targets for glioma drug development.

## Materials and methods

### Cell lines

Human glioma cell line U87 was sourced from Cobioer Biosciences Co, Ltd (Nanjing, China), and normal human astrocytes (NHAs) were from Sciencell Research Laboratories (Carlsbad, USA). U87 cells were kept in DMEM containing 10% serum and 1% penicillin-streptomycin. NHAs were cultured in astrocyte medium (Life Technologies) [23].

### Cell transfection and processing

miR-125a-5p mimic/inhibitor and its negative control miR-NC, the overexpression plasmid for TXLNA (pcDNA3.1-TXLNA) and the negative control (pcDNA3.1-vector) were provided by GenePharma (Shanghai, China). For transient transfection, Lipofectamine 3000 (Invitrogen, USA) was used. The effectiveness of transfection after 24 h was assessed by RT-qPCR and Western blot.

U87 cells were digested with trypsin to prepare single-cell suspensions and inoculated in 96-well plates at 1 × 10^4^ /well, with three replicate wells set up in each group. ATOR (Dr. Abidi Company, Iran) was dissolved in 0.1% dimethyl sulfoxide/PBS solution (w/v). After the cells adhered to the wall, the wells were treated with culture solution containing different concentrations of ATOR (0, 5, 10, 15, 20 µM; Sigma-Aldrich) for 24 h [24].

### Dual luciferase assay

starBase 3.0 (http://starbase.sysu.edu.cn/) predicted the potential binding sites of miR-125a-5p and TXLNA. GenePharma (Shanghai, China) synthesised wild-type (wild-type) or mutant (mutant) TXLNA containing the binding site of miR-125a-5p, which were cloned into the pmirGLO luciferase reporter vector (Promega, USA) to construct wild-type (TXLNA-WT) and mutant reporters (TXLNA-MUT). Using Lipofectamine^®^ 3000 (Invitrogen), U87 cells were inoculated into 96-well plates at 1 × 10^4^ cells per well. The cells were then co-transfected with a luciferase reporter plasmid containing either miR-125a-5p mimic or mimic-NC. 48 h later, the Dual Luciferase Assay System (Promega) identified the luciferase reporter plasmid.

### RNA immunoprecipitation (RIP) assay

RIP assay for protein binding of miR-125a-5p and TXLNA was performed using the Magna RIP Kit (Millipore). U87 cells underwent lysis using RIP lysis buffer, followed by co-incubation of 10 µL of the cell lysates with magnetic beads infused with anti-Ago2 (Abcam, USA) or anti-IgG (Abcam), attached to these beads, for 6 h at 4 °C. The immunoprecipitates attached to magnetic beads were extracted, followed by an analysis of miR-125a-5p and TXLNA through RT-qPCR post RNA purification.

### Cell viability assay

U87 cells underwent inoculation in 96-well plates at 5 × 10^3^ and were incubated for 12 h. Following a 24-hour treatment period, each well received 20 µl of 5 mg/ml MTT solution for 4 h at 37 °C. After removing the medium, the insoluble metazan crystals were dissolved in 150 µl of dimethyl sulfoxide (Sigma-Aldrich). A microplate reader was used to track the absorbance at 490 nm (Sanco, China).

### PCR

Total RNA was extracted from tissues and U87 cells using Trizol reagent (Thermo) and RNA concentration was measured using a bicinchoninic acid kit (Invitrogen). The miRNA reverse transcription kit (TaKaRa) facilitated the creation of cDNA from miRNAs, while the PrimeScriptTM RT Reagent kit (TaKaRa) was employed for mRNA synthesis. For each specimen, PCR was conducted utilizing the SYBR green PCR premix kit (Invitrogen) on a CFX96 contact real-time fluorescent quantitative PCR detection system (Bio-Rad, CA, USA). RNA expression was determined by employing the 2^−ΔΔCt^ technique. Table 1 displays the sequences of the primers.

**Table 1:** Primer sequence

Gene	Forward primer (5′ →3′)	Reverse primer (5′ →3′)
miR−125a−5p	TCCCTGAGACCCTTTAAC	TTTGGCACTAGCACATT
TXLNA	AAAGCCAAGGGTTTGGGGAA	CCTCTGGGACTCTACTGCCT
GAPDH	CCCAGCAAGAGCACAAGAGGAAG	GAGGGGAGATTCAGTGTGGTGGG

### Western blot

Using RIPA lysis buffer (FD008, Vazyme), U87 cells and tissues underwent lysis on ice for 20 min. The Pierce ALI Protein Assay Kit (Rockford) was employed to measure protein levels. Proteins underwent separation through 10% SDS-PAGE and were then moved onto PVDF membranes (Millipore). The membranes underwent closure using 5% skimmed milk for two hours, followed by individual incubation with the primary antibody: TXLNA (Proteintech. 18243-1-AP), GAPDH (Abcam; ab37168), Bax (Abcam; ab32503), Bcl-2 (Abcam; ab182858), PD-L1 (Abcam; ab205921), PKM1 (Abcepta; C-term L398), and PKM2 (FineTest; FNab06496) overnight at 4 °C, and combined for 1 h at 37 °C with secondary antibody (Abcam; ab205719). Finally, the results were visualized with the ECL Detection Kit (Vazyme; E411-04) and the results were checked on the FluorChem™M system.

### Colony formation assay

U87 cells were inoculated into 6-well plates with 500 cells per well. DMEM containing 10% foetal bovine serum was added and cultured for a fortnight. After clonal colony formation was observed, the medium was removed. Cells were fixed with 75% ethanol (Beyotime) for 10 min, stained with 0.1% crystal violet (Sigma) for 10 min, and imaged through a microscope (Olympus, Japan).

### Transwell experiments

Transwell chambers (BD Biosciences, USA) treated with matrigel (BD Biosciences) were equipped with a pore size of 8 μm (Corning, NY, USA), whereas no matrigel was used in the migration experiments. U87 cells (5 × 10^4^ migration/8 × 10^4^ invasion) were resuspended in the upper chamber with 200 µL serum-free medium, and 600 µL medium supplemented with 10% foetal calf serum (HyClone) was added to the lower chamber. After 24 h, cells in the upper chamber were removed, fixed in the lower chamber with 4% paraformaldehyde (Beyotime), imaged with a microscope (Olympus), and analyzed with ImagU87 software.

### Flow cytometry

The rate of apoptosis was evaluated using the Annexin V-FITC/PI Apoptosis Detection kit (Invitrogen). A total of 1 × 10^5^ cells were reconstituted in 500 µl of 1 × Binding Buffer, followed by the addition of 5 µl of Annexin V-FITC and propidium iodide in that order. A FACScan^®^ flow cytometer (BD Biosciences, USA) was employed to assess the proportion of apoptotic cells following a 30-minute staining process.

### Assessment of glycolytic biomarkers

With the Glucose Assay Kit (Betoyime), Lactate Assay Kit (Sigma) and ATP Detection Kit (Sigma), glucose uptake, lactate secretion, and ATP production were determined in U87 cell culture medium.

### Co-culture system

PBMCs from healthy individuals were separated using Lymphoprep density gradient centrifugation (07851, Stemcell). To enhance T cell activation, PBMCs underwent cultivation using U87 cell lysate (0.5 mg/ml) and IL-2 (20 ng/ml) in 6-well plates, with each well containing 3 × 10^6^ cells for 72 h. Following stimulation, PBMCs were collected and cleansed using Lymphoprep density gradient centrifugation, then jointly cultured with U87 cells at 10:1 for 16 h. T cells expressing CD8 were isolated using CD8 magnetic beads (Miltenyi, 130-096-730). TNF-α and IFN-γ in the co-culture medium were measured through ELISA (ThermoFisher).

### Flow cytometry

Activated T cells were co-cultured with U87 cells at an effector/target (E/T) ratio of 5:1 for 24 h. The percentage of apoptosis of U87 cells was detected using flow cytometry and Annexin V-APC/PI Apoptosis Kit (KeyGEN BioTECH).

The concentration of U87 cells was calibrated to 1 × 10^5^/100 µl, followed by a 30-minute incubation with IFN-γ-APC (BioLegend, 502511), TNF-α-APC (BioLegend, 502913), and mouse IgG1-APC (BioLegend, 400119), using centrifugation at 900 r/min. Ultimately, the supernatant was extracted, followed by flow cytometry to measure IFN-γ and TNF-α in CD8T [22]. The outcomes of flow cytometry were examined utilizing the FlowJo software.

### Nude mouse xenograft

All animal studies were performed according to protocols approved by the Zibo Central Hospital Animal Care and Use Committee. Twenty 5-week-old BALB/c nude mice (18–22 g, National Collection of Cell Culture, Shanghai, China) were established for xenograft model assays. U87 cells were resuspended in saline, and a suspension of U87 cells (5 × 10^5^ cells, 100 µL) was injected subcutaneously into the right neck of nude mice. Drug-treated mice were administered 10 mg/kg ATOR intraperitoneally, while control mice were administered intraperitoneally using 0.1% dimethyl sulfoxide (DMSO)/PBS solution (w/v). Administration of ATOR was initiated at the same time as the injection of U87 cells. After 4 days of injection, miR-125a-5p agomir (RiboBio, Guangzhou, China) or negative control (10 nmol) was injected into tumors every 4 days for 28 days. Mice were housed in a pathogen-free environment with a 12-hour light/dark cycle, temperature of 22 ± 3°C, and relative humidity of 50% ± 15%. A vernier caliper was used to measure the tumor’s length and width every four days. The tumor’s volume was computed using the formula tumor volume (mm^3^) = (short diameter)^2^ × (long diameter) × 0.5. The mice died due to cervical dislocation, and tumor tissue was collected for subsequent analysis.

### HE staining

Tumor samples underwent fixation in 4% paraformaldehyde for a day, followed by dehydration, embedding in paraffin, slicing into 5 μm coronal sections, and staining with HE staining (Beyotime). The sections underwent differentiation using 1% hydrochloric acid mixed in alcohol for 10 min, followed by a 10-second rinse with 2% sodium bicarbonate (Beyotime), and then a 3-minute staining with eosin. Post-dehydration using a series of alcohol concentrations, the samples were cleansed using xylene and then encased in neutral resin. A Tokyo Olympus BX 53 microscope was employed to observe pathological alterations.

### Immunohistochemistry (IHC)

Samples of tumor tissue from nude mice were preserved in 4% paraformaldehyde, dehydrated using an ethanol solution, and then encased in paraffin. Tissues encased in paraffin were sliced to a thickness of 3 μm and then arranged on slides. After deparaffinisation in xylene (Beyotime), the tissue sections were blocked with 0.3% H_2_O_2_ for 10 min, and incubated with PBS containing 5% FBS and 0.3% Triton X-100 for 1 h. TXLNA (Proteintech. 18243-1-AP), PKM2 (FineTest; FNab06496), and PD-L1 (Abcam; ab205921) antibodies were incubated overnight at 4 °C, as well as secondary antibodies (cab6720, Abcam) for 1 h. Target signals were developed by DAB substrate (Vector Labs, Burlingame, USA) and the slides were re-stained with hematoxylin for 2 min and visualized using a microscope (Leica).

### Data analysis

Statistical analysis of the experimental data was conducted utilizing the SPSS20 statistical program. The data presentation was in the form of an average ± standard deviation. A t-test was conducted to compare two groups. For comparisons between two sets of data, one-way analysis of variance (ANOVA) was employed. For comparisons between multiple data sets, two-way analysis of variance (ANOVA) was conducted. All experiments were replicated at least three times, and histogram results were expressed as the mean + standard deviation of three biological replicates (five biological replicates for animal experiments) in a typical experiment. * *P <* 0.05 signifies a statistical difference.

## Results

### ATOR inhibits proliferation and induces apoptosis in glioma cells

**Fig. 1:**
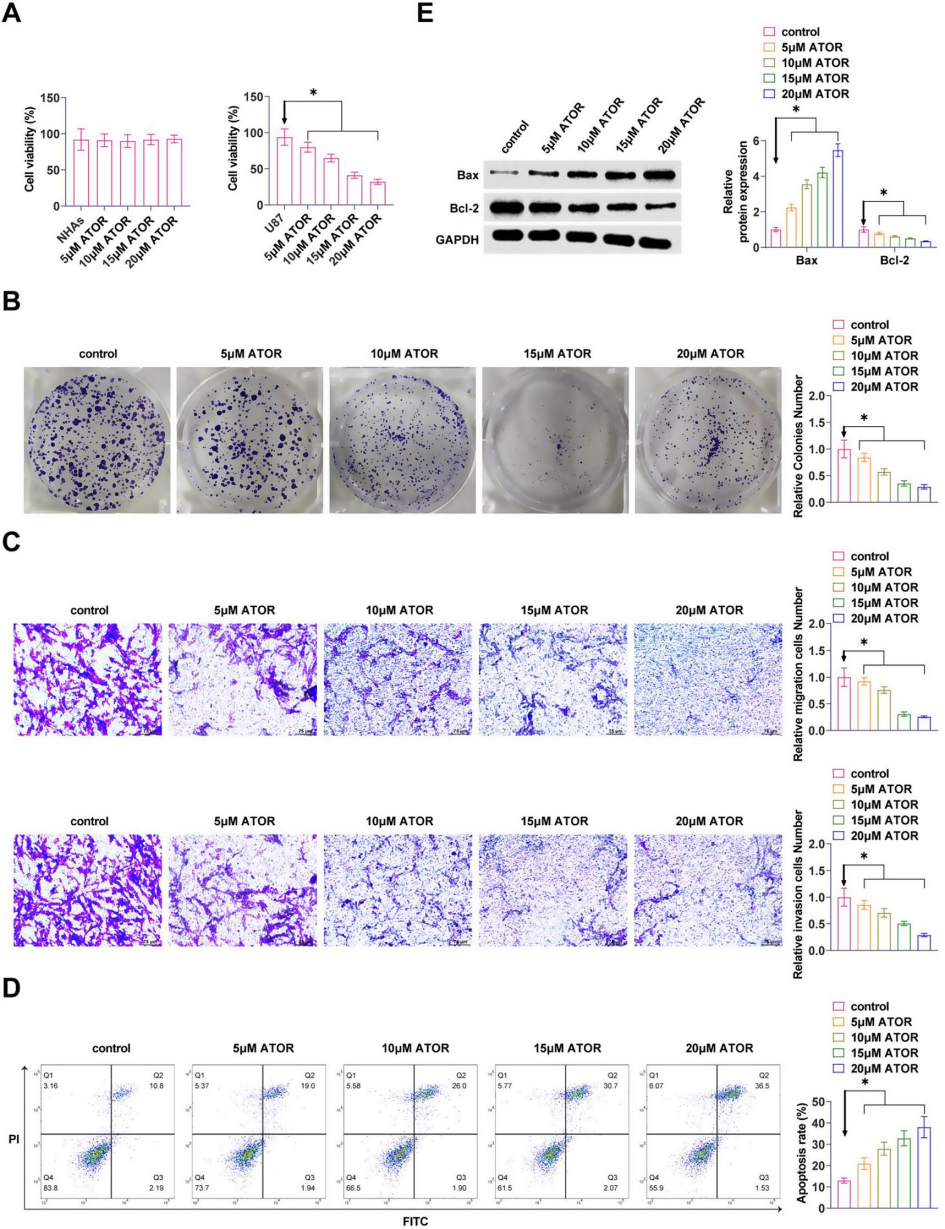
ATOR inhibits proliferation and induces apoptosis in glioma cells. **A**: MTT assay to detect the effects of different doses of ATOR on the proliferative ability of U87 cells; **B**: Colony formation assay to detect the effects of different doses of ATOR on the proliferative ability of U87 cells; **C**: Transwell assay to detect the effects of different doses of ATOR on the migratory and invasive ability of U87 cells; **D**: Flow cytometry to detect the effects of different doses of ATOR on the apoptotic level of U87 cells; **E**: Western Blot assay to detect the effects of different doses of ATOR on the expression of Bax and Bcl-2 proteins in U87 cells. Data are expressed as mean ± SD (*n* = 3). * *P* < 0.05

U87 cells were exposed to different doses of ATOR (0, 5, 10, 15, 20 µM). MTT assay reported that ATOR significantly reduced cellular proliferative capacity in a dose-dependent manner, but had no effect on the viability of NHAs (Fig. 1A). Meanwhile the results of colony formation assay manifested that ATOR significantly reduced the proliferation ability of U87 cells (Fig. 1B). Transwell assay exhibited that ATOR repressed the migration and invasion ability of U87 cells (Fig. 1C). Detection by flow cytometry exhibited that ATOR promoted the apoptosis level of U87 cells (Fig. 1D). Western Blot assay exhibited that ATOR promoted Bax and inhibited Bcl-2 expressions in U87 cells (Fig. 1E). The effect of ATOR was dose-dependent.

### ATOR inhibits glycolysis and immune escape in glioma cells

**Fig. 2:**
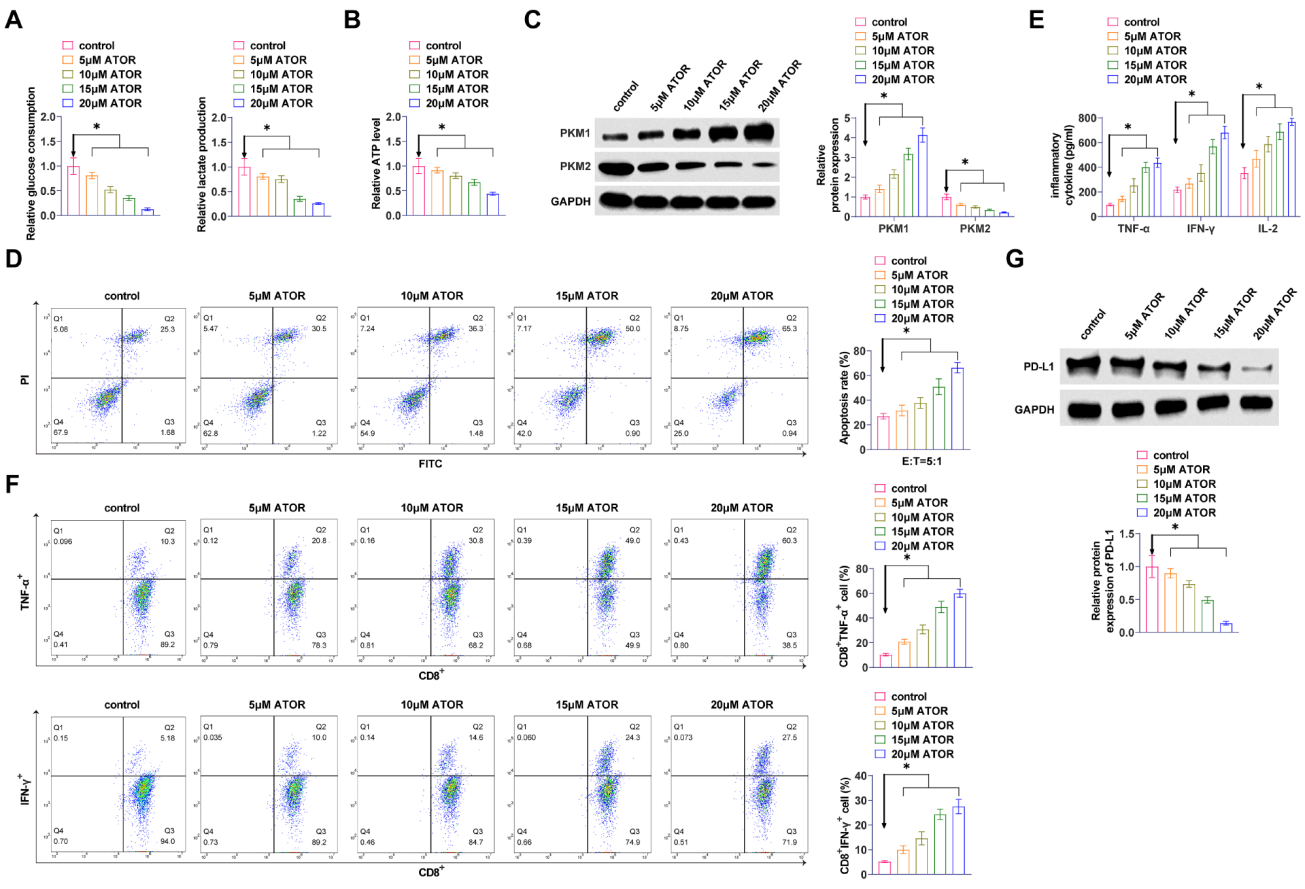
ATOR inhibits glycolysis and immune escape in glioma cells. **A**: Effect of ATOR on glucose consumption and lactate production in U87 cells; **B**: Effect of ATOR on ATP production in U87 cells; **C**: Western Blot to detect the effect of ATOR on PKM1 and PKM2 in U87 cells; **D**: Flow cytometry to study the effect of ATOR on T cell-mediated apoptosis in U87 cells; **E**: ELISA to detect the effect of ATOR on the TNF-α, IFN-γ, and IL-2 secretion in cell cultures; **F**: Flow cytometry to detect the effect of ATOR on TNF-α and IFN-γ production in CD8^+^ T cells; **G**: Western Blot to detect the effect of ATOR on PD-L1 expression in U87 cells. Data are expressed as mean ± SD (*n* = 3). * *P* < 0.05

ATOR reduced glucose consumption, lactate production, and ATP production in U87 cells dose-dependently (Fig. 2A, B). Western Blot assay exhibited that ATOR enhanced PKM1 and inhibited PKM2 in U87 cells (Fig. 2C). ATOR increased the proportion of apoptotic U87 cells after CD8^+^ T cell culture (Fig. 2D). The secretion of TNF-α, IFN-γ, and IL-2 in cell cultures was significantly and dose-dependently increased by ATOR (Fig. 2E). CD8^+^ T cells were isolated, and flow cytometry revealed that TNF-α and IFN-γ positive populations in CD8 + T cells increased with the rise in the concentration of ATOR (Fig. 2F). Western Blot assay determined that ATOR inhibited PD-L1 expression (Fig. 2G). ATOR at 15 µM reduced cell viability to approximately 50% and was therefore selected for subsequent experiments.

### Silencing miR-125a-5p reduces the effect of ATOR on glioma proliferation and apoptosis

**Fig. 3:**
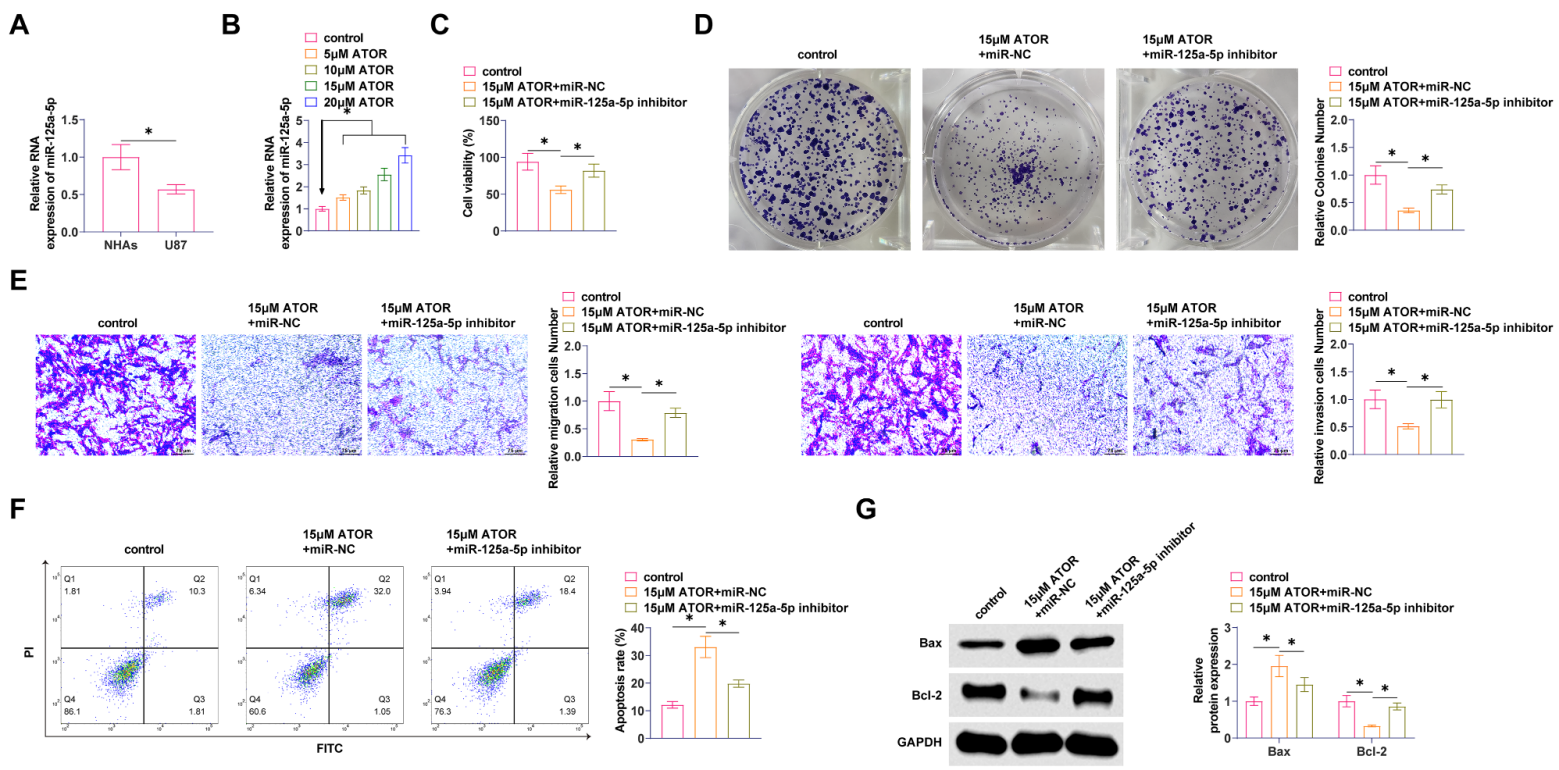
Silencing miR-125a-5p reduces the effect of ATOR on glioma proliferation and apoptosis miR-125a-5p inhibitor or miR-NC was transfected into U87 cells. **A**: RT-qPCR to detect miR-125a-5p; **B**: RT-qPCR to detect the effect of ATOR on miR-125a-5p; **C**: MTT assay to determine the viability of U87 cells; **D**: Colony formation assay to determine the proliferation of U87 cells; **E**: Transwell assay to assess the migration and invasion ability of U87 cells; **F**: Flow cytometry to determine the apoptosis rate of U87 cells; **G**: Western Blot to detect Bax and Bcl-2 in U87 cells. Data are expressed as mean ± SD (*n* = 3). * *P* < 0.01

RT-qPCR found that miR-125a-5p in U87 cells was significantly lower than that in NHAs (Fig. 3A). Meanwhile, miR-125a-5p in U87 expression increased with ATOR concentration (Fig. 3B). Cell viability of U87 cells was significantly increased after knockdown of miR-125a-5p, which weakened the ATOR effect (Fig. 3C, D). ATOR was impaired by miR-125a-5p inhibition in its ability to inhibit migration and invasion of U87 cells (Fig. 3E). Also, down-regulating miR-125a-5p significantly inhibited apoptosis in U87 cells (Fig. 3F). ATOR promoted Bax and inhibited Bcl-2 proteins in U87 cells, and miR-125a-5p inhibitor prevented this effect (Fig. 3G).

### Silencing miR-125a-5p weakens the effect of ATOR on glycolysis and immune escape in glioma

**Fig. 4:**
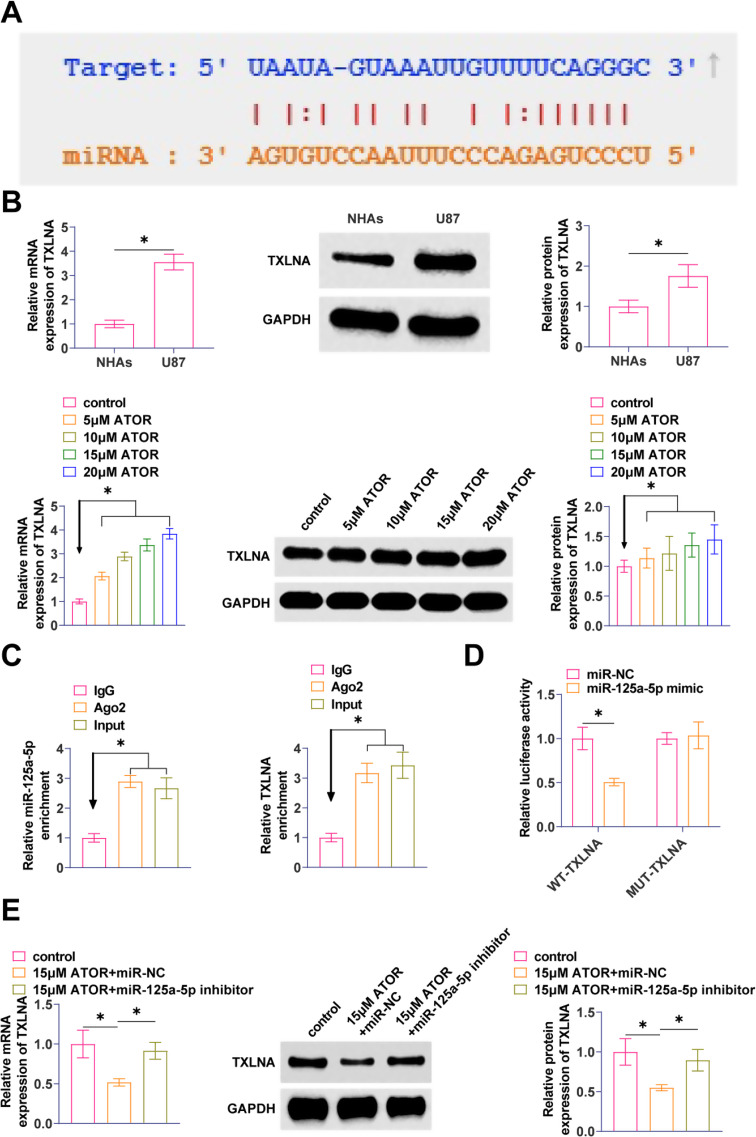
Silencing miR-125a-5p weakens the effect of ATOR on glycolysis and immune escape in glioma. **A**: Glucose consumption and lactate production in U87 cells; **B**: ATP production in U87 cells; **C**: Western Blot detection of PKM1 and PKM2 expression in U87 cells; **D**: Flow cytometry study of T-cell-mediated apoptosis in U87 cells; **E**: ELISA to detect TNF-α, IFN-γ and IL- 2 secretion; **F**: Flow cytometry to detect TNF-α and IFN-γ production in CD8^+^ T cells; G: Western Blot to detect PD-L1 expression in U87 cells. Data are expressed as mean ± SD (*N* = 3). * *P* < 0.05

A reduction in miR-125a-5p led to a significant increase in glucose consumption, lactate production, and ATP output in U87 cells (Fig. 4A, B), reversing the effects of ATOR. Moreover, silencing miR-125a-5p inhibited PKM1 and promoted PKM2 in U87 cells (Fig. 4C). We subsequently investigated the effect of ATOR on immune escape in glioma cells. Knocking down miR-125a-5p impeded the promotional effect of ATOR on the apoptotic ratio of U87 cells after CD8^+^ T cell culture (Fig. 4D), and the secretion of TNF-α, IFN-γ, and IL-2 (Fig. 4E). A significant increase in the number of TNF- and IFN-positive CD8 + T cells was observed due to ATOR action, but miR-125a-5p downregulation negated that effect (Fig. 4F). Also, miR-125a-5p inhibition promoted PD-L1 expression (Fig. 4G).

### TXLNA is a target gene of miR-125a-5p

**Fig. 5:**
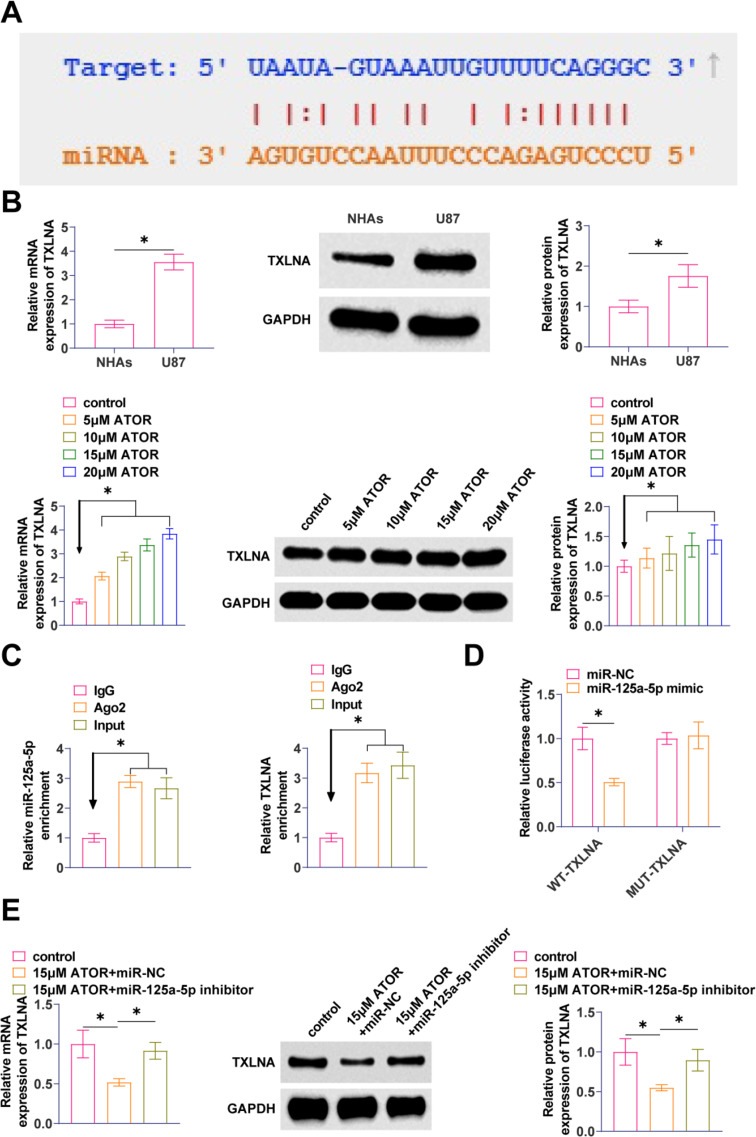
TXLNA is a target gene of miR-125a-5p **A**: starBase 3.0 predicted the binding site of miR-125a-5p to TXLNA; **B**: RT-qPCR and Western Blot to detect TXLNA in U87 cells; **C**: RIP assay to determine the binding effect of miR-125a-5p to TXLNA; **D**: Dual-luciferase reporter assay to detect the binding relationship between miR-125a 5p and TXLNA; E: RT-qPCR and Western Blot to detect TXLNA in U87 cells after transfection with miR-125a-5p inhibitor. Data are expressed as mean ± SD (*N* = 3). * *P* < 0.01

The molecular target of miR-125a-5p was found to be TXLNA with a possible binding site as predicted by the bioinformatics website starBase3.0 (Fig. 5A). TXLNA in gliomas was elevated, but TXLNA expression decreased with increasing ATOR concentration (Fig. 5B). Ago2 immunomagnetic beads significantly enriched in TXLNA and miR-125a-5p by RIP analysis (Fig. 5C). By co-transfecting miR-125a-5p and WT-TXLNA, significant inhibition of luciferase activity was observed after co-transfection of miR-125a-5p and WT-TXLNA, demonstrating the validity of the interaction between these two factors (Fig. 5D). RT-qPCR and Western Blot assays exhibited that suppressing miR-125a-5p enhanced TXLNA expression in U87 cells (Fig. 5E).

### TXLNA overexpression enhances the effect of down-regulating miR-125a-5p on ATOR-regulated glioma cells

**Fig. 6:**
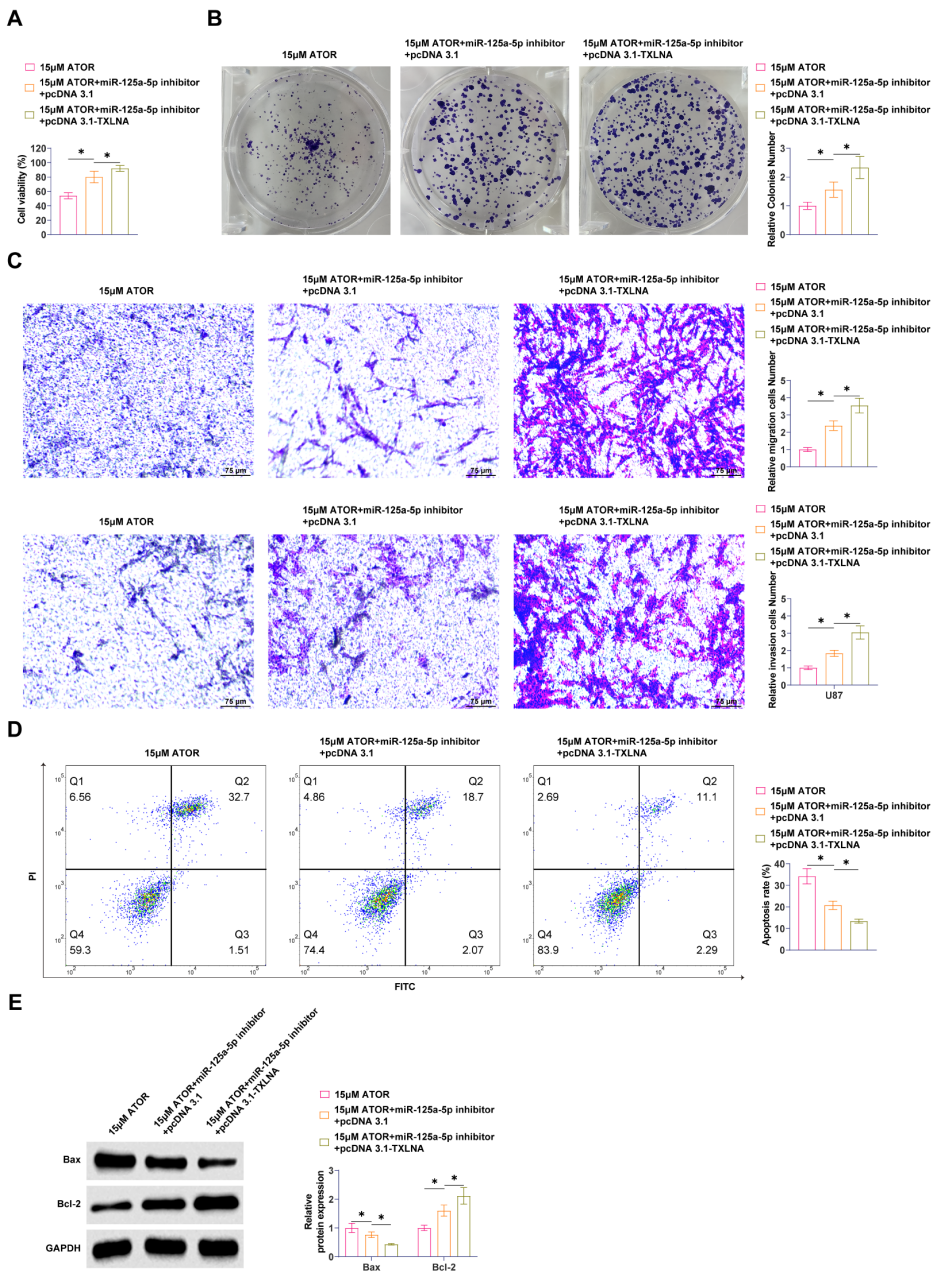
TXLNA overexpression enhances the effect of down-regulating miR-125a-5p on ATOR-regulated glioma cells. pcDNA3.1-TXLNA and miR-125a-5p inhibitor were co-transfected into U87 cells at the same time of ATOR administration. **A**: MTT assay to determine the viability of U87 cells; **B**: Colony formation assay to determine the proliferation ability of U87 cells; **C**: Transwell assay to assess the migration and invasion ability of U87 cells; **D**: Flow cytometry to determine the apoptosis rate of U87 cells; **E**: Western Blot to detect Bax and Bcl-2 in U87 cells. Data are expressed as mean ± SD (*N* = 3). * *P* < 0.05

In parallel with ATOR administration, pcDNA3.1-TXLNA and miR-125a-5p inhibitor were co-transfected into U87 cells for loss-of-function assay. Enhancing TXLNA promoted U87 cell viability and proliferation significantly (Fig. 6A, B). Reducing miR-125a-5p impaired the effects of ATOR on U87 cell migration and invasive ability, while the intervention of overexpressing TXLNA further enhanced the effect of downregulating miR-125a-5p (Fig. 6C). Overexpression of TXLNA significantly inhibited apoptosis of U87 cells and enhanced the effect of downregulating miR-125a-5p (Fig. 6D). ATOR promoted Bax and inhibited Bcl-2 proteins in U87 cells, and elevating TXLNA further enhanced the reducing miR-125a-5p on the reversal of ATOR (Fig. 6E).

### Overexpression of TXLNA promotes the effect of downregulating miR-125a-5p on ATOR-regulated glycolysis and immune escape in glioma

**Fig. 7:**
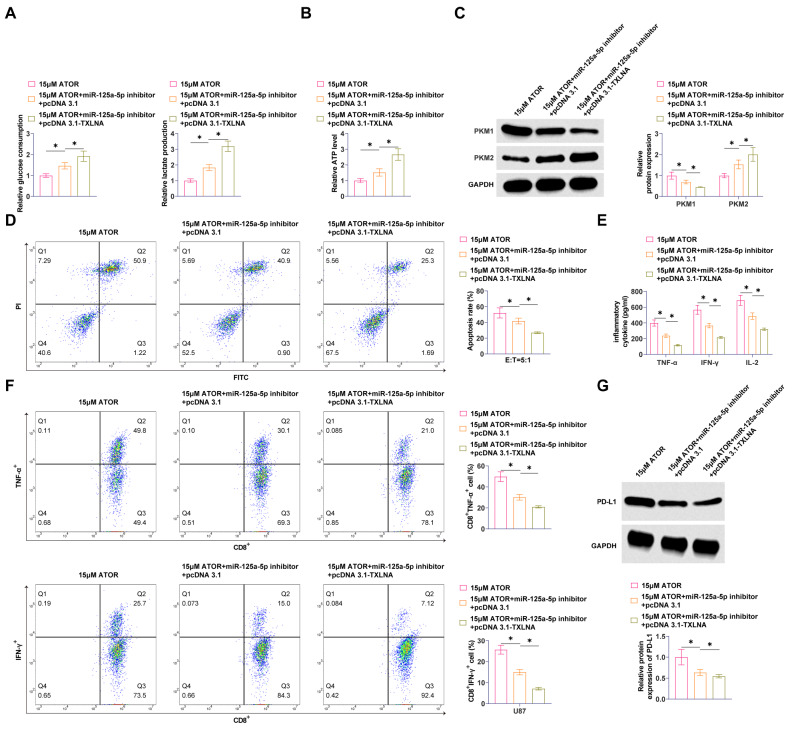
Overexpression of TXLNA promotes the effect of downregulation of miR-125a-5p on ATOR-regulated glycolysis and immune escape in glioma. **A**: Glucose consumption and lactate production in U87 cells; **B**: ATP production in U87 cells; **C**: Western Blot detection of PKM1 and PKM2 expression in U87 cells; **D**: Flow cytometry study of T-cell-mediated apoptosis in U87 cells; **E**: ELISA to detect TNF-α, IFN-γ and IL-2 secretion; **F**: Flow cytometry to detect TNF-α and IFN-γ production in CD8^+^ T cells; **G**: Western Blot to detect PD-L1 expression in U87 cells. Data are expressed as mean ± SD (*N* = 3). * *P* < 0.05

ATOR inhibition of glucose consumption, lactate production, and ATP production in U87 cells was blocked by silencing miR-125a-5p, while overexpression of TXLNA enhanced the effects of miR-125a-5p reduction (Fig. 7A, B). miR-125a-5p inhibitor blocked the effect of ATOR on PKM1 and PKM2 expression, and overexpression of TXLNA further enhanced the effect of down-regulated miR-125a-5p to inhibit PKM1 and promote PKM2 levels (Fig. 7C). Subsequently, reducing miR-125a-5p blocked the promotion of ATOR on the apoptotic ratio of U87 cells cultured with CD8^+^ T cells, and overexpression of TXLNA augmented this effect (Fig. 7D). The secretion of TNF-α, IFN-γ, and IL-2 in the culture medium was significantly elevated by ATOR, while reducing miR-125a-5p blocked the effect, and overexpression of TXLNA further augmented the impact of reducing miR-125a-5p (Fig. 7E). ATOR promoted TNF-α and IFN-γ-positive populations in CD8^+^ T cells, and overexpression of TXLNA further augmented the reversal of ATOR by downregulation of miR-125a-5p (Fig. 7F). miR-125a-5p inhibitor blocked the inhibition of PD-L1 expression by ATOR, and overexpression of TXLNA enhanced the reversal of down-regulated miR-125a-5p (Fig. 7G).

### ATOR regulates miR-125a-5p expression to inhibit glioma development

**Fig. 8:**
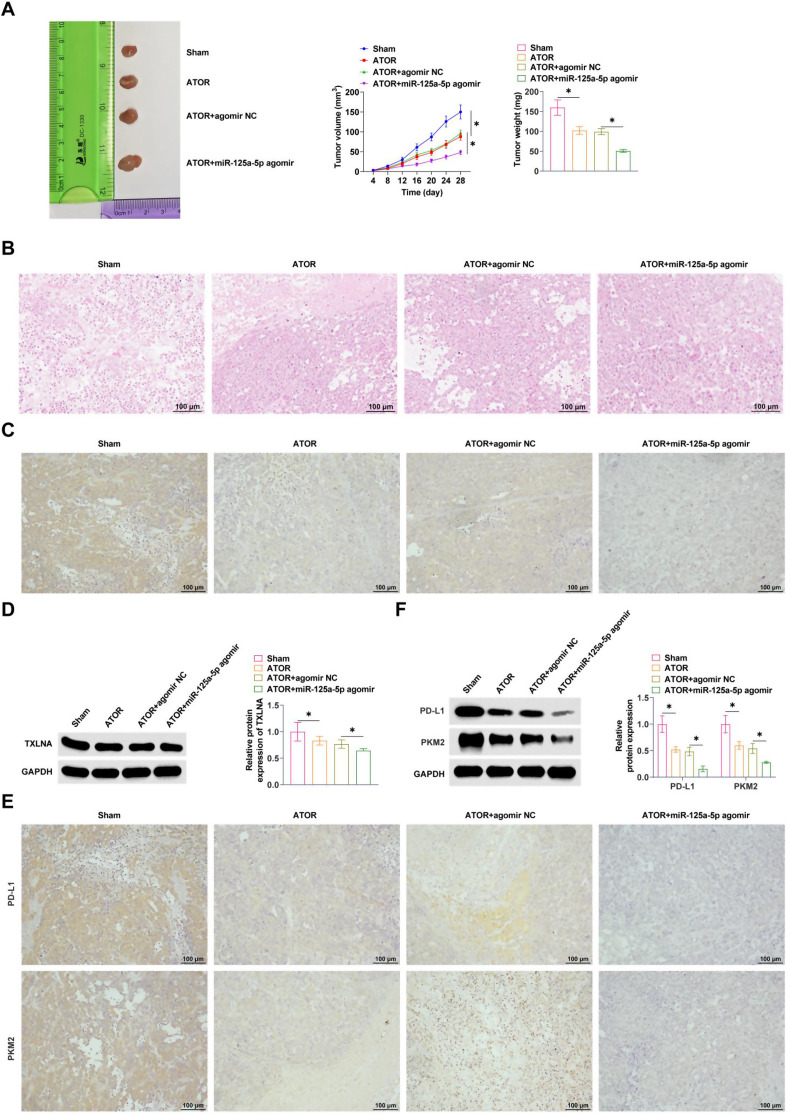
ATOR regulates miR-125a-5p expression to inhibit glioma development. **A**: Tumor growth; **B**: HE staining to detect tumor pathology; **C**: IHC staining to determine the expression of TXLNA; **D**: Western Blot to detect the expression of TXLNA; **E**: IHC staining to determine the expression of PD-L1 and PKM2; **F**: Western Blot to detect PD-L1 and PKM2 expression. Data are expressed as mean ± SD (*n* = 5). * *P* < 0.05

The effect of ATOR on tumor growth was first examined by transplanting the tumor model in nude mice. ATOR significantly delayed tumor growth, reduced tumor volume and weight, and miR-125a-5p agomir enhanced the effect of ATOR (Fig. 8A). HE staining exhibited that the tumor was solid, irregular and without peritoneum, and the tumor foci were seen to have irregular morphology and unclear boundaries, and clusters of tumor cells were seen in the surrounding normal brain tissue cell infiltration, abundant blood vessels in the tumor tissue, and haemorrhage and necrosis were seen in the tumor centre. ATOR treatment significantly improved the tumor histopathology, while miR-125a-5p agomir enhanced the effect of ATOR (Fig. 8B). In addition, IHC staining exhibited that ATOR inhibited TXLNA expression, which was further inhibited by miR-125a-5p agomir (Fig. 8C). Western Blot exhibited consistent results (Fig. 8D). IHC assay exhibited that ATOR inhibited PKM2 and PD-L1 in tumor tissues (Fig. 8E). Similarly, Western Blot assay exhibited that ATOR treatment inhibited PD-L1, PKM2, and miR-125a-5p agomir treatment further promoted this effect (Fig. 8F).

## Discussion

Radiation therapy, regular chemotherapy, and tumor removal are the main treatments for primary gliomas [25]. ATOR is a new lipid-soluble statin that can cross the blood-brain barrier, which is conducive to the practical application in the clinical treatment of glioma. The present study elucidates a new mechanism that may be effective in the treatment of gliomas. ATOR inhibits tumor glycolysis and immune escape through miR-125a-5p-mediated regulation of TXLNA, thereby contributing to the alleviation of glioma progression.

Previous studies have confirmed the ability of ATOR to inhibit Bcl-2 and Caspase-3 expression thereby inhibiting glioma angiogenesis and ultimately inhibiting glioma cell progression [26]. It suggests that ATOR effectively inhibits glioma development. In addition to this, previous studies have not found that ATOR regulates the miR-125a-5p/TXLNA axis in other diseases. The biological behaviors involved in malignant lesions of glioma cells mainly include apoptosis, proliferation, invasion and metastasis, which are multi-linked and complex biological processes. Based on these properties of glioma, we first explored the specific mechanisms of ATOR for the treatment of glioma. In this study, we selected different doses of ATOR for our experiments, mainly considering the efficacy, safety, and tolerance of different doses. In our preexperiment, the choice of 5–20 µM better reflects the differences in the efficacy of different doses of ATOR on glioma. This is in general agreement with a previous study by Oliveira et al. [24]. In this study, ATOR treatment significantly inhibited progression of glioma cells. More importantly, it was shown that ATOR treatment inhibited glycolysis in gliomas by detecting glucose consumption, lactate production, and ATP generation. Pyruvate kinase plays a crucial role in cellular metabolism and PKM1 and PKM2 are isoforms present in mammals. It has been shown that only PKM2 is expressed in cancer cells and that PKM2 expression is significantly increased in glioma cells [27] and is higher than that of PKM1. This is because PKM2 can be localized to the nucleus through phosphorylation and post-translational modifications, thus acting as a transcription factor to promote tumor cell growth and survival. Similarly, ATOR increased the production of TNF-α and IFN-γ, inhibited PD-L1, and suppressed immune escape from gliomas. Among them, IFN-γ is the main stimulator of PD-L1 expression [28]. Tumor-penetrating T cells emit IFN-γ, which plays a twofold function in the study of cancer immunity. This cytokine serves dual roles: firstly, as an anti-tumor agent essential for eradicating tumors, and secondly, as tumor cells persistently subjected to IFN-γ demonstrate enhanced immune evasion capabilities [29]. Thus, our data reveal a new drug that modulates PD-L1 expression. Notably, potential side effects of ATOR in the clinical setting include muscle pain and damage, liver damage, elevated blood glucose or type 2 diabetes, and neurological side effects, especially at high doses. ATOR is often co-administered with other medications for therapeutic purposes. For example, the combination of ATOR with cardiovascular medications can further lower blood pressure and reduce the incidence of cardiovascular events [30], and the combination of ATOR with antiplatelet agents can reduce the formation of atherosclerotic plaques and reduce the risk of thrombosis [31]. Overall, the synergistic effect of ATOR with other therapies is mainly in the enhancement of the therapeutic effect. However, the specific synergistic effects need to be determined on a patient-by-patient basis.

Dysregulation of the tumor-associated miRNA network plays a crucial role in gliomas. In a previous study, reducing miR-125a-5p promotes the onset of glycolysis in gastric cancer cells [32]. In gliomas, miR-125a-5p expression was downregulated and increased after ATOR treatment. Depleting miR-125a-5p impeded the effects of ATOR on gliomas, and promoted glycolysis and immune escape in glioma cells. Alterations in miRNA regulation participate in glioma through oncogene and oncogene regulation mechanism, which in turn affects downstream signaling pathways [33–35]. Rescue experimental results exhibited that suppressing miR-125a-5p impaired the inhibitory effects of ATOR on glioma cells, while overexpressing TXLNA enhanced the effects of suppressing miR-125a-5p on glioma cells.

There are some limitations of this study. Immunosuppressive factors in the tumor microenvironment, such as regulatory T cells and tumor-associated macrophages, need to be taken into account when studying the immune escape of glioma cells. This suggests that the dynamics of immune escape is important to improve the accuracy and reliability of the study results. However, due to the limitations of the sample size and animal model, an in-depth exploration of immune escape was lacking in this study. In future studies, we will expand the sample size and specify reasonable and reliable study protocols to further investigate the dynamic changes of immune escape in vivo.

This study confirmed that inhibition of tumor glycolysis and immune escape by the miR-125a-5p/TXLNA axis is critical for suppressing tumor cell proliferation and progression. ATOR therapy was further explored, and the molecular function of miR-125a-5p/TXLNA in tumor progression was greatly expanded. In addition, this study provides data to better understand tumor formation and progression, and hopefully contributes to the discovery of new therapeutic targets.

## Data Availability

Data is available from the corresponding author on request.
